# In vivo impact of JAK3 A573V mutation revealed using zebrafish

**DOI:** 10.1007/s00018-022-04361-8

**Published:** 2022-05-27

**Authors:** Faiza Basheer, Vilasha Bulleeraz, Viet Q. T. Ngo, Clifford Liongue, Alister C. Ward

**Affiliations:** 1 School of Medicine, Deakin University, Pigdons Road, Geelong, VIC 3216 Australia; 2 IMPACT, Deakin University, Geelong, VIC Australia

**Keywords:** Lymphocytes, Leukemia, JAK3, Cytokine receptor signaling, Zebrafish

## Abstract

**Background:**

Janus kinase 3 (JAK3) acts downstream of the interleukin-2 (IL-2) receptor family to play a pivotal role in the regulation of lymphoid cell development. Activating JAK3 mutations are associated with a number of lymphoid and other malignancies, with mutations within the regulatory pseudokinase domain common.

**Methods:**

The pseudokinase domain mutations A572V and A573V were separately introduced into the highly conserved zebrafish Jak3 and transiently expressed in cell lines and zebrafish embryos to examine their activity and impact on early T cells. Genome editing was subsequently used to introduce the A573V mutation into the zebrafish genome to study the effects of JAK3 activation on lymphoid cells in a physiologically relevant context throughout the life-course.

**Results:**

Zebrafish Jak3 A573V produced the strongest activation of downstream STAT5 in vitro and elicited a significant increase in T cells in zebrafish embryos. Zebrafish carrying just a single copy of the Jak3 A573V allele displayed elevated embryonic T cells, which continued into adulthood. Hematopoietic precursors and NK cells were also increased, but not B cells. The lymphoproliferative effects of Jak3 A573V in embryos was shown to be dependent on zebrafish IL-2Rγc, JAK1 and STAT5B equivalents, and could be suppressed with the JAK3 inhibitor Tofacitinib.

**Conclusions:**

This study demonstrates that a single JAK3 A573V allele expressed from the endogenous locus was able to enhance lymphopoiesis throughout the life-course, which was mediated via an IL-2Rγc/JAK1/JAK3/STAT5 signaling pathway and was sensitive to Tofacitinib. This extends our understanding of oncogenic JAK3 mutations and creates a novel model to underpin further translational investigations.

**Supplementary Information:**

The online version contains supplementary material available at 10.1007/s00018-022-04361-8.

## Background

Cytokine receptor signaling via the Janus kinase-Signal transducer and activator of transcription (JAK-STAT) pathway represents a core mode of regulation in the context of blood and immune cell development and function [50, 58]. Of the four mammalian JAKs, JAK3 is unique in having restricted expression in hematopoietic cells, particularly within the lymphoid compartment [18]. JAK3 exclusively associates with the common interleukin-2 receptor gamma common (IL-2Rγc) chain that is shared by the receptors of several cytokines critical for lymphopoiesis, specifically IL-2, IL-4, IL-7, IL-9, IL-15 and IL-21 [57]. JAK1 has been shown to be critical for signaling from these receptors [20, 54], which occurs via several intracellular signaling pathways, but particularly STAT5 [34, 43].

The importance of JAK3 in normal lymphopoiesis is illustrated by inactivating mutations that cause autosomal Severe Combined Immune Deficiency (SCID) that is characterized by reduced T and NK cells as well as non-functional B cells, mediated by loss of function of the suite of IL-2Rγc-utilizing receptors [10]. In contrast, constitutively activating JAK3 mutations—variously impacting the FERM, SH2, pseudokinase and kinase domains—have been detected in in several cases of human hematopoietic neoplasms, specifically cutaneous T cell lymphoma (CTCL) [11, 45], intestinal T cell lymphoma [49], T cell acute lymphoblastic leukemia (T-ALL) [3, 48], T cell prolymphocytic leukemia (T-PLL) [6], Natural killer T cell lymphoma (NKTCL) [27], as well as acute megakaryoblastic leukemia (AMKL) [25, 56, 64]. Two common mutations within the pseudokinase domain (PKD), A572V and A573V, have been identified in NKTCL [8, 27], T-ALL [3, 17], intestinal T cell lymphoma [49], T-PLL [6] and AMKL [44, 64]. These mutations cause JAK3 to be constitutively activated and able to mediate ligand-independent proliferation of cells expressing them, including a range of lymphoid cells [27, 64]. JAK1 has been shown to be essential for JAK3 mutants to mediate their effects [17, 54].

Zebrafish show strong conservation of cytokine receptor signaling through the JAK-STAT pathway [37, 38], including a conserved role for the IL-2Rγc in immune cell development [59, 60]. This study examined the A572V and A573V JAK3 PKD mutations, which were introduced into zebrafish Jak3 and the effects on STAT5 activation in vitro and T lymphocyte production in vivo analyzed. A mutant zebrafish *jak3* allele encoding the Jak3 A573V mutant was generated by genome editing, with fish carrying one or two alleles analyzed for immune cell development from embryonic through to adult stages. Genetic analysis was used to assess the importance of IL-2Rγc, JAK1 and STAT5 in mediating the effects of the JAK3 A573V mutation, and sensitivity to the JAK3 inhibitor Tofacitinib analyzed. Collectively, this work provides critical in vivo insights into oncogenic JAK3.

## Methods

### In vitro analyses

Human HEK293T cells were transfected with pBKCMV expressing HA-tagged zebrafish Jak3 wild type [60] as well as A572V and A573V mutants (synthesized by Genewiz LLC), along with zebrafish Stat5.1 [32] and analyzed by Western blot with anti-phospho-STAT5, anti-STAT5 and anti-GAPDH, as described [60].

### Fish husbandry and genetic manipulations

Zebrafish were maintained using standard husbandry practices [65], following national guidelines for their care and use, with all studies approved by the Deakin University Animal Ethics Committee. Wild-type embryos at the 1 cell stage were injected with 100 pg/μl in vitro transcribed, capped mRNA encoding zebrafish Jak3 A572V and A573V mutants. For CRISPR/Cas9 genome editing, a guide RNA that targeted exon 13 of the *jak3* gene was produced using a specific primer pair (5′-TAGGAGATTTGACTCATCAAAC and 5′-AAACGTTTGATGAGTCAAATCT), as described [21]. Wild-type embryos were injected with 12.5 ng/μl gRNA, 100 ng/μl Cas9 mRNA (Sigma) and 10 μM homology-dependent repair (HDR) oligonucleotide (5’-TCTTCTTTTTTCTAGTCTCTTTTCGAGGCGGTATCCTTGATGAGTCAAATCTCCCACAGGCACCTTC), raised to adulthood and outcrossed with wild-type fish. Carriers of the *jak3* mutant allele were identified and, following an additional round of outcrossing, were in-crossed to generate wild-type, heterozygote and homozygote mutant progeny for analysis. The *jak3* mutant was also crossed onto lines carrying hypomorphic mutations in *il2rga* [59], as well as *jak1*, *stat5.1* and *stat5.2* produced in-house (unpublished data) to generate additional genotypes for analysis.

### Genomic DNA analysis

Genomic DNA from adult fin clips and whole embryos was isolated with QuickExtract following the manufacturer’s instructions. This was subjected to PCR with *jak3*-specific primers, either for restriction fragment length polymorphism (RFLP) analysis with *Bci*VI (5′-TTATCCATGTGAATAAATGTTTAATCTTC, 5′-CTAATGCCATACACCAAAAGAAGG) or High Resolution Melt (HRM) (5′-TTATCCATGTGAATAAATGTTTAATCTTC, 5′-CTAATGCCATACACCAAAAGAAGG) using Precision Melt Suremix and Analysis Software (BioRad) to identify *jak3* mutants. These were individually confirmed by Sanger sequencing at the Australian Genome Research Facility. Genotyping details for the other gene mutations is included in the relevant Supplementary Figures.

### RT-PCR and qRT2-PCR

Total RNA was extracted from individual 28 dpf juvenile zebrafish with RNeasy Mini Kit (Qiagen) according to the manufacturer’s protocol for animal tissues. This was subjected to semi-quantitative reverse-transcription polymerase chain reaction (RT-PCR) with primers for T-cell receptor beta (TCRβ) variable chains *vb1.5/17.5* (5′-AATGGACAGCTTGATAGAACTGAAC, 5′-TGCTTATTCAACCGAACAGAAACATTC), *vb12* (5′-CAGACACCGTGCTTCAGTCGAG, 5′-ACGTTTCATGGCAGTGTTACCTG) and *vb14.5* (5′-GAATCCAATGTGACGTTAACATGC, 5′-CATGATCATAAGGACCACTACAG) and immunoglobulin variable heavy chains *igvh1* (5′-GATGGACGTGTTACAATTTGG, 5′-CCTCCTCAGACTCTGTGGTGA) and *igvh4* (5′-CAAGATGAAGAATGCTCTCTG, 5′-TGTCAAAGTATGGAGTCGA) or quantitative real-time RT-PCR (qRT^2^-PCR) with *actb* (5′-TGGCATCACACCTTCTAC, 5′-AGACCATCACCAGAGTCC), *cmyb* (5′-TCGGCAAGACACGCTGGA, 5′-AATGCTTTGCGATTACTGACCA), *rag1* (5′-GGATGTGAAGTATGTGTGTTTGA, 5′-TGGAACCCAGGGAGAAGC), *cd4* (5′-TCTTGCTTGTTGCATTCGCC, 5′-TCCCTTTGGCTGTTTGTTATTGT), *cd8* (5′-ACTCTTCTTCGGAGAGGTGAC, 5-ACAGGCTTCAGTGTTGTTTGAA), *cd79a* (5′-GCGAGGGTGTGAAAAACAGT, 5-CCCTTTCTGTCTTCCTGTCCA), *igm* (5′-CCGAATACAGTGCCACAAGC, 5′-TCTCCCTGCTATCTTTCCGC), *nccrp1* (5′-TCAGCACAGGTGGTTCACTCTA, 5′-GGCTTTCCTCATACCAGTCTTC) and *nkld* (5′-TGGTGAAATCCCAACAGAGCA, 5′-TTTCATCCTGAGTTGCACCA). Data were normalized to *actb* and fold change was calculated using the ΔΔCt method.

### Whole-mount in situ hybridization (WISH) and hemoglobin staining

Anesthetized embryos were dechorionated and fixed in 4% (w/v) paraformaldehyde at 4°C before WISH with anti-sense digoxigenin-labeled gene-specific probes, as described (Thisse and [62], or subjected to staining of hemoglobin with *O*-dianisidine [35]. Quantitation was achieved by enumeration of individual cells or measuring the area of staining relative to eye diameter using CellSens Dimension 1.6 software (Olympus) in a blind fashion on images taken on an Olympus MVX10 monozoom microscope with a 1 × MVXPlan Apochromat lens (NA = 0.25) with an Olympus DP72 camera. Data from approximately 30 embryos were analyzed for significance with a Student’s *t* test using Welch's correction where necessary, with data tested for normality.

### Ex vivo analyses

Cytospin preparations were prepared from embryonic and adult blood as well as adult kidney and stained with Giemsa (Sigma), and differential counts performed. These were imaged on a Leica DM E microscope with a 100 × oil objective (NA = 1.25) with an Olympus SC50 camera Alternatively, adult zebrafish kidney cells were prepared in ice-cold phosphate-buffered saline supplemented with 2 mM EDTA and 2% (v/v) fetal calf serum and passaged through a 40 μm sieve and analyzed using a FACSCantoII with cell populations identified in a side-scatter (SSC)/forward-scatter (FSC) plot as described [4]. Data were analyzed for significance with a Student’s *t* test.

### Survival analysis

Survival of juvenile/adult fish was monitored by regular visual inspection and displayed as a Kaplan–Meier curve with statistical significance determined using a log-rank (Mantel–Cox) test.

## Results

### Analysis of zebrafish Jak3 mutants

**Fig. 1:**
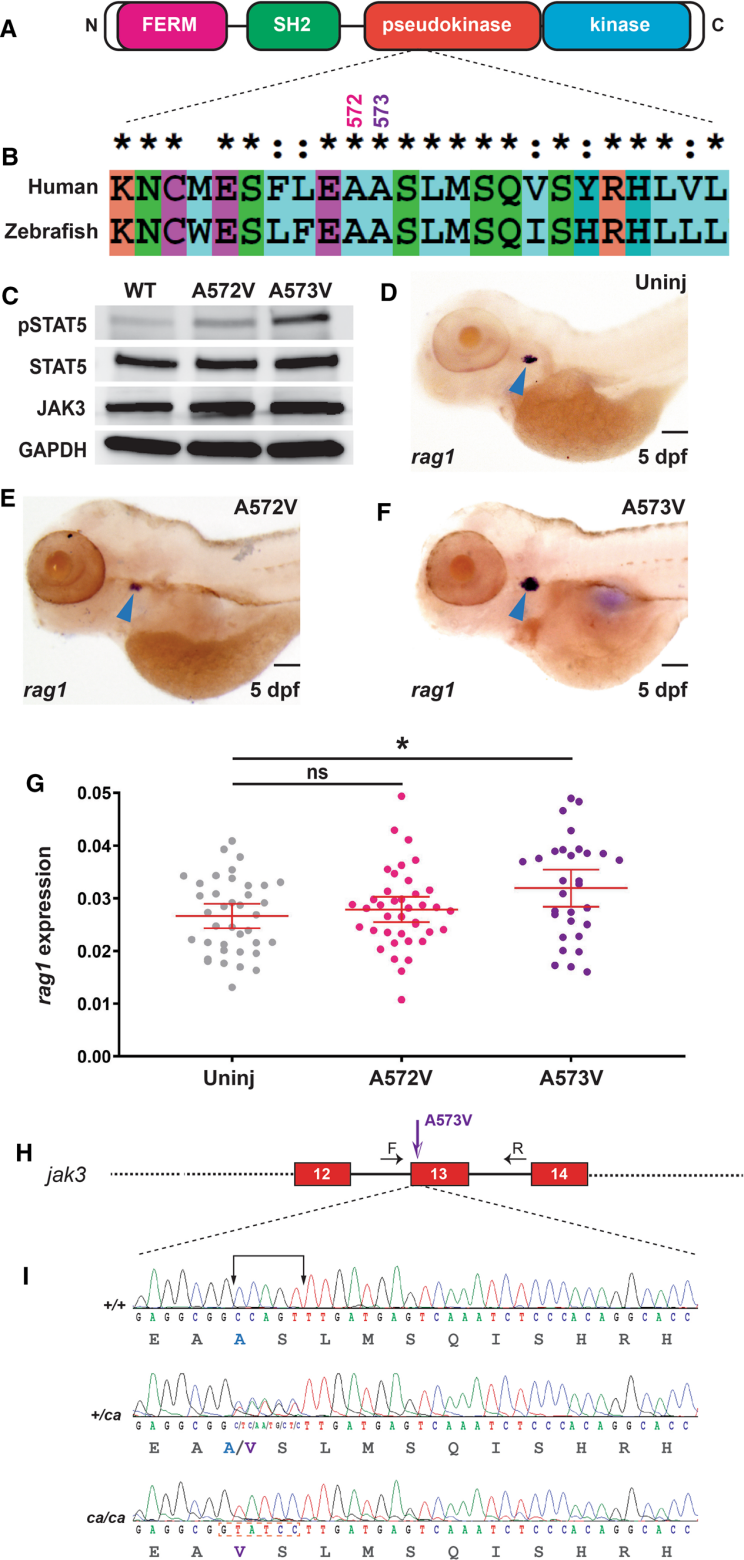
Conservation of human JAK3 constitutively-activating mutations in zebrafish. **A** JAK3 structure. Schematic representation of JAK3 including the FERM (pink), SH2 (green), pseudokinase (red) and kinase (blue) domains, which are all present in zebrafish Jak3. **B** Conservation of human and zebrafish JAK3. Human JAK3 and zebrafish Jak3 were aligned using CLUSTALX, showing identical (*) and highly similar (:) residues around A572/A573. **C** Analysis of zebrafish Jak3 mutants in vitro. Western blot analysis of HEK293 cells transfected with wild-type Jak3 + Stat5.1 (WT), Jak3 A572V + Stat5.1 (A572V) and Jak3 A573V + Stat5.1 (A573V) using anti-phospho-STAT5 (pSTAT5), anti-STAT5, anti-HA (JAK3) and anti-GAPDH antibodies, as indicated. **D**–**G** Analysis of zebrafish Jak3 mutants in vivo. Uninjected (Uninj) embryos (**D**) or those injected with mRNA encoding Jak3 A572V (**E**) or A573V (**F**), were subjected to WISH with *rag1* and imaged, with representative embryos shown. The area of *rag1* expression was determined for individual embryos (**G**) with mean and SEM shown in red and level of statistical significance indicated (*p < 0.05, ns: not significant; *n* = 30) (6.3 × magnification, scale bar = 100 μm). **H** Genome targeting of zebrafish *jak3*. Exon–intron structure of the target site with spanning primers (**F**, **R**) indicated by black arrows, with exons encoding the pseudokinase domain shown as numbered boxes and introns represented as solid lines, with the Jak3 A573V mutation denoted by a purple arrow. **I** The *jak3* constitutively activating (ca) mutant alleles generated in zebrafish. The nucleotide sequence of homozygote wild-type (+*/*+), along with heterozygote (+*/ca*) and homozygote (*ca/ca*) mutant zebrafish are shown with the five bases mutated indicated and the introduced *Bci*VI site boxed. The protein translations are shown below in black text, with the exception of wild-type A573 in blue and mutant V573 in purple

Zebrafish possess a highly conserved Jak3 [37] that contains the same domain structure as human JAK3 (Fig. 1A), and high amino acid identity extending to residues within the PKD, including those encompassing A572 and A573 (Fig. 1B). To analyze the effect of A572V and A573V mutations in the context of zebrafish Jak3, expression constructs encoding these variants and wild-type Jak3 tagged with HA were transfected into human HEK293 cells along with one encoding zebrafish Stat5.1, with Stat5.1 activation determined by Western blot using an anti-phospho-STAT5 antibody (Fig. 1C). Modest phospho-STAT5 was observed with Jak3 wild-type, as described [60], which was increased with Jak3 A572V and even more so with Jak3 A573V. Analysis with anti-STAT5, anti-HA and anti-GAPDH antibodies confirmed equivalent transfection and loading in each case (Fig. 1C). To confirm these effects in vivo*,* one-cell stage wild-type zebrafish embryos were injected with in vitro transcribed mRNA encoding Jak3 A572V or A573V and at 5 days post-fertilization (dpf) were subjected to WISH with *rag1* as a marker of mature T lymphocytes [67]. This revealed increased expression of *rag1* in embryos injected with Jak3 A573V, but not Jak3 A572V, compared to control uninjected embryos (Fig. 1D–G).

### Generation of zebrafish carrying T cell lymphoma-derived Jak3 mutation

Since the zebrafish Jak3 A573V mutant elicited robust Stat5.1 activation in vitro and increased T cells in vivo, it was selected for further study. To introduce this mutation in the zebrafish genome, a CRISPR/Cas9 HDR strategy was used [61]. Guide RNA targeting exon 13 was co-injected with Cas9-encoding mRNA along with a 67 bp single-stranded oligonucleotide repair template that contained missense nucleotide changes in the coding sequence to elicit a A573V mutation, as well as silent changes to introduce a restriction enzyme site (*Bci*VI) to facilitate screening and destroy the protospacer adjacent motif to prevent further recognition by Cas9 (Fig. 1H–I). Injected embryos were raised to adulthood for screening, which identified zebrafish carrying an allele encoding the constitutively activating Jak3 A573V mutant (designated *ca*). These were outcrossed for two generations to remove any potential off-target mutations, followed by an in-cross to generate heterozygote Jak3 A573V (+*/ca*) mutants, mimicking patient mutations, as well as homozygote (*ca/ca*) mutants, which were confirmed by sequencing (Fig. 1I).

### Impact of Jak3 A573V mutation on embryonic lymphoid cell development

**Fig. 2:**
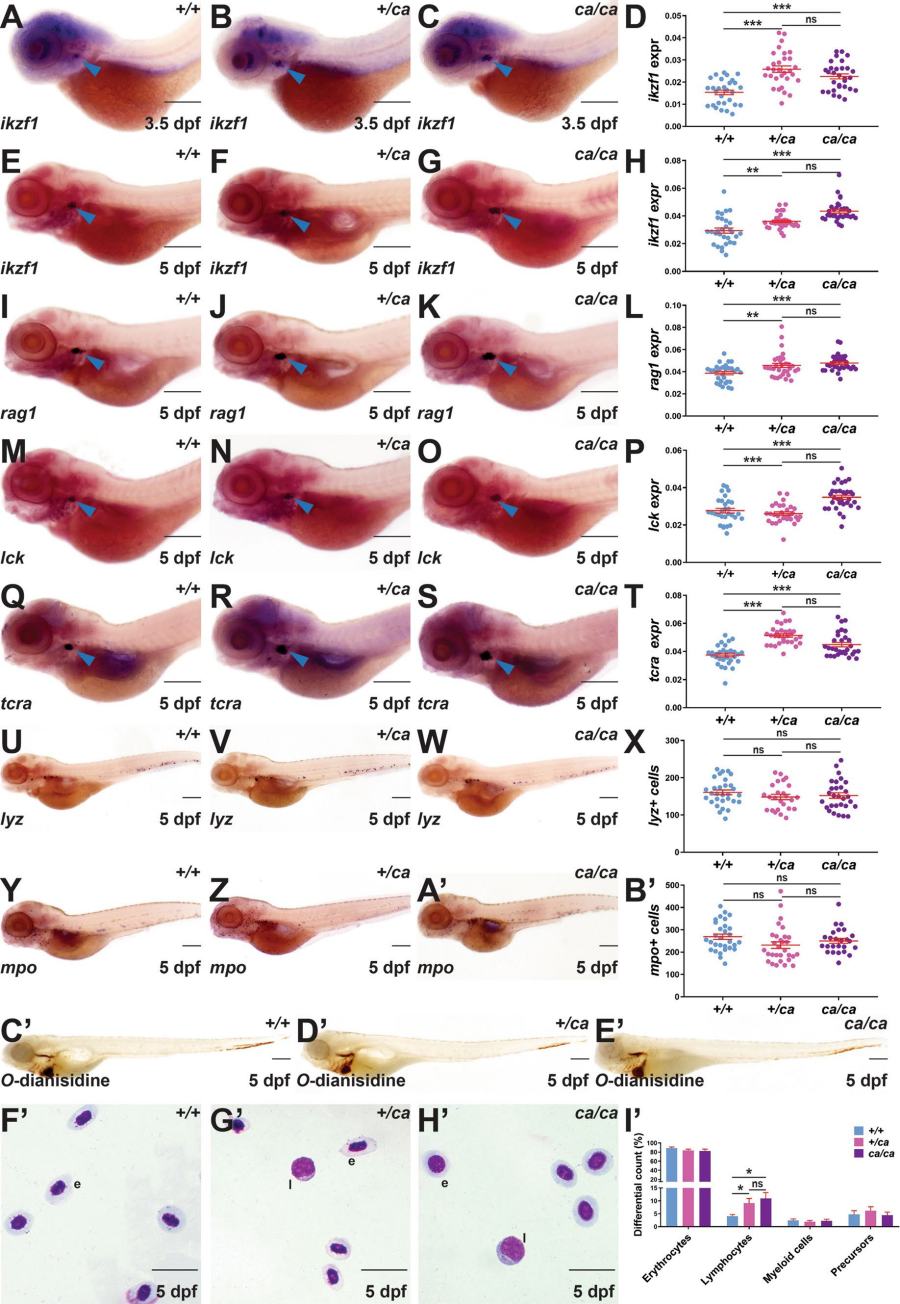
Phenotypic analysis of zebrafish carrying the Jak3 A573V encoding allele. Embryos homozygous for Jak3 wild­type (*jak3*^+*/*+^) or Jak3 A573V (*jak3*^*ca/ca*^) or  those heterozygous for Jak3 A573V (*jak3*^+*/ca*^) were subjected to WISH with *ikzf1* at 3.5 dpf (**A**–**C**) and 5 dpf (**E**–**G**), *rag1* (**I**–**K**), *lck* (**M**–**O**), *tcra* (**Q**–**S**), *lyz* (**U**–**W**) and *mpo* (**Y**–**A**′) at 5 dpf, as well as *O-*dianisidine staining (**C**′–**E**′) (6.3/3.2 × magnification, scale bar = 200 μm) or their blood analyzed with Giemsa staining at 5 dpf (**F**′–**H**′; e, erythrocyte, l, lymphocyte) (100 × magnification, scale bar = 10 μm). Individual embryos were assessed for relative area of staining with *ikzf1* (**D, H**), *rag1* (**L**), *lck* (**P**) and *tcrα* (**T**), for individual embryos or total number of *lyz+ *^+^(**X**) and *mpo+* (**B**′) cells, or blood differential counts (**I**′) with the mean and SEM shown in red and level of statistical significance indicated (*p < 0.05, **p < 0.01, ***p < 0.001, ns: not significant) (*n* = 30 for WISH analysis and *n* = 5 for blood counts)

Lymphocyte progenitors first populate the zebrafish thymus from approximately 2.5 dpf to initiate T lymphopoiesis [29]. To investigate the effect of the Jak3 A573V mutation on embryonic lymphopoiesis, wild type (*jak3*^+*/*+^) along with heterozygote (*jak3*^+*/ca*^), and homozygote (*jak3*^*ca/ca*^) mutants, were subjected to WISH with the early lymphoid marker *ikzf1* [66], as well as the mature lymphocyte markers *rag1* [67], *lck* [28] and *tcra* [13]. Both *jak3*^+*/ca*^ and *jak3*^*ca/ca*^ embryos showed a significant increase in *ikzf1* expression compared to *jak3*^+*/*+^ embryos at both 3.5 dpf (Fig. 2A–D) and 5 dpf (Fig. 2E–H), with a similar increase also evident in the expression of *rag1* (Fig. 2I–L), *lck* (Fig. 2M-P) and *tcra* (Fig. 2Q–T) at 5 dpf. Interestingly, no significant differences were observed between *jak3*^+*/ca*^ and *jak3*^*ca/ca*^ embryos (Fig. 2D, H, L, P, T). In contrast, the number of *lyz*+ leucocytes [39] (Fig. 2U–X), *mpo*+ neutrophils [35] (Fig. 2Y–B′) and the extent of *O*-dianisidine staining of erythrocytes [35] (Fig. 2C′–E′) was not significantly different between *jak3*^+*/*+^, *jak3*^+*/ca*^ and *jak3*^*ca/ca*^ embryos. Analysis of blood smears at 5 dpf confirmed a significant increase in the proportion of lymphocytes in *jak3*^+*/ca*^ and *jak3*^*ca/ca*^ embryos in comparison to their *jak3*^+*/*+^ siblings, but again no statistically significant difference between *jak3*^+*/ca*^ and *jak3*^*ca/ca*^ embryos was observed (Fig. 2F′–I′).

### Analysis of zebrafish harboring the Jak3 A573V mutation into adulthood

**Fig. 3:**
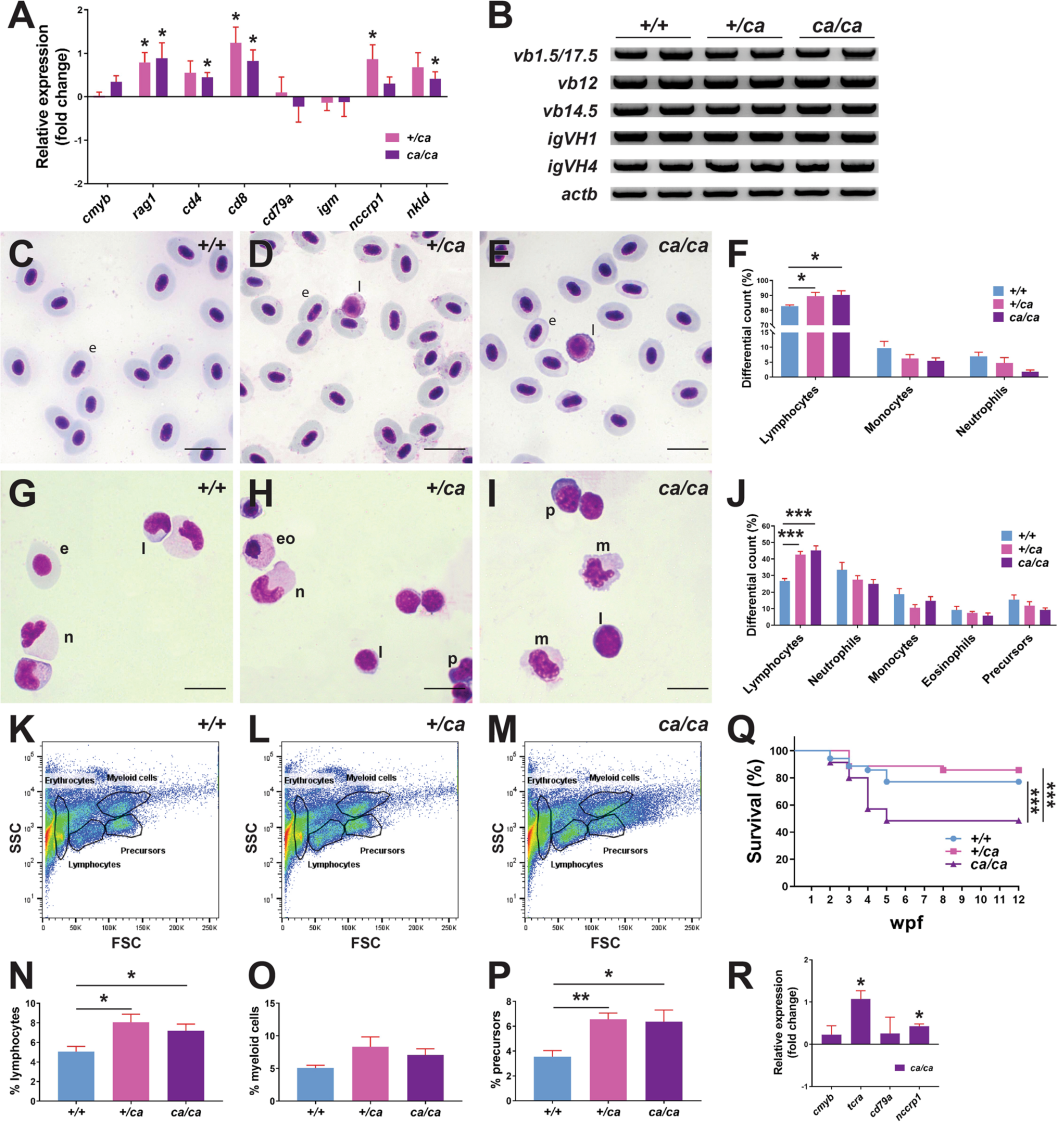
Effect of Jak3 A573V mutation on later hematopoiesis. **A**, **B** Gene expression analysis. Total RNA extracted from 28 dpf homozygous wild-type (*jak3*^+*/*+^)*,* heterozygote (*jak3*^+*/ca*^) and homozygote (*jak3*^*ca/ca*^) mutant zebrafish was subjected to qRT^2^­PCR analysis (**A**) with gene markers of hematopoietic stem cells (*cmyb*), T cells (*rag1, cd4, cd8*), B cells (*cd79a, igm*), and NK cells (*nccrp1, nkld*). Data are represented as relative fold change compared to homozygous wild-type (+*/*+) fish, with mean and SEM shown in red and statistical significance compared for Cq values normalized to control *actb* indicated (*p < 0.05; *n* = 3), or to RT-PCR (B) with primers specific for T cell receptor (TCR) β-chain (v(*d*)*j­cβ vb1.5*, *vb12*, *vb14.5*) and B cell Ig heavy chain (*igVH1*, *igVH4*) rearrangements and *actb* as the control and analyzed by agarose gel electrophoresis. RT­-negative controls yielded no products (data not shown) (*n* = 2). **C**–**J** Histological analysis. Giemsa stained blood cells (**C–E**) and kidney cells (**G–I**) of adult wild-type (*jak3*^+*/*+^) (**C, G**), heterozygote (*jak3*^+*/ca*^) (**D, H**) and homozygote (*jak3*^*ca/ca*^) (**E, I**) fish, with differential counts for blood (**F**) and kidney (**J**) performed (e: erythrocyte, eo: eosinophil, l: lymphocyte, n: neutrophil, p: precursor, m: macrophage) (*n* = 4–8; 100 × magnification; scale bar = 10 μm). **K–P** FACS analysis. Kidney cells from adult wild-type (*jak3*^+*/*+^) (K)*,* heterozygote (*jak3*^+*/ca*^) (**L**) and homozygote (*jak3*^*ca/ca*^) (**M**) were subjected to FACS analysis, with lymphoid (**N**) myeloid (**O**) and precursor (**P**) populations quantified, with mean and SEM shown in red and level of statistical significance indicated (*p < 0.05, **p < 0.01; *n* = 5). **Q** Survival analysis. Wild-type (*jak3*^+*/*+^)*,* heterozygote (*jak3*^+*/ca*^) and homozygote (*jak3*^*ca/ca*^) zebrafish were assessed weekly for survival, which is displayed as a Kaplan–Meier plot with level of statistical significance indicated (***p < 0.001, *n* = 35). **R** Gene expression analysis. Total RNA extracted from adult homozygous wild-type (*jak3*^+*/*+^)*,* heterozygote (*jak3*^+*/ca*^) and homozygote (*jak3*^*ca/ca*^) zebrafish was subjected to qRT^2^­PCR analysis with gene markers of hematopoietic stem cells (*cmyb*), T cells (*tcra*), B cells (*cd79a*), and NK cells (*nccrp1*), as described for panel **A**

Zebrafish B lymphocytes develop from around 3 weeks post-fertilization [51], with NK-related cells also readily detectable at this time [46]. To examine the effects of Jak3 A573V mutation on these lineages, qRT^2^-PCR was performed on 28 dpf *jak3*^+*/*+^, *jak3*^+*/ca*^ and *jak3*^*ca/ca*^ larvae using primers specific for genes marking HSCs (*cmyb*), T cells (*rag1*, *cd4*, *cd8*), B cells (*cd79a*, *igm*) and NK cells (*nccrp1*, *nkld*) [46]. This revealed that both *jak3*^+*/ca*^ and *jak3*^*ca/ca*^ larvae displayed increased expression of T cell markers, reaching significance for *rag1* and *cd8* in both and *cd4* in *jak3*^*ca/ca*^ larvae, and NK cell markers, reaching significance for *nccrp1* in *jak3*^+*/ca*^ and *nkld* in *jak3*^*ca/ca*^ larvae, whereas markers of HSCs and B cells were not significantly altered (Fig. 3A). Both *jak3*^+*/ca*^ and *jak3*^*ca/ca*^ larvae showed normal T cell and B cell rearrangement (Fig. 3B).

Adult *jak3*^+*/*+^, *jak3*^+*/ca*^ and *jak3*^*ca/ca*^ zebrafish were also investigated. Cytological analysis of blood (Fig. 3C–E) and its quantitation by differential counting (Fig. 3F) revealed a significant increase in circulating lymphocyte numbers in *jak3*^+*/ca*^ and *jak3*^*ca/ca*^ in comparison to *jak3*^+*/*+^ fish. Analysis of the adult kidney, which serves a similar function to mammalian bone marrow with respect to hematopoiesis [7], identified a significant increase in lymphocyte numbers in both *jak3*^+*/ca*^ and *jak3*^*ca/ca*^ in comparison to *jak3*^+*/*+^ fish (Fig. 3G-J). Kidney cells were also subjected to flow cytometry analysis, with the lymphocyte (Fig. 3K-M, N) and precursor (Fig. 3K-M, P) populations both increased significantly in *jak3*^+*/ca*^ and *jak3*^*ca/ca*^ in comparison to *jak3*^+*/*+^ fish. In contrast, the myeloid (Fig. 3K-M, O) populations showed no statistically significant alterations. Expression analysis confirmed increased *tcra*+ T cells and *nccrp1*+ NK cells in this tissue in male fish (Fig. 3R).

The survival of *jak3*^+*/*+^, *jak3*^+*/ca*^ and *jak3*^*ca/ca*^ fish was also monitored. Mortality of *jak3*^*ca/ca*^ fish was increased during the first 5 wpf compared to *jak3*^+*/*+^ and *jak3*^+*/ca*^ fish (Fig. 3Q), but this stabilized such that these fish reached adulthood and were healthy and fecund (data not shown).

### Molecular analysis of Jak3 A573V mutants

Other research has implicated various cytokine receptor signaling components in mediating the effects of JAK3 and mutants thereof [17, 20, 41]. The possession of a number of relevant zebrafish mutants made it possible to analyze these interactions genetically.

**Fig. 4:**
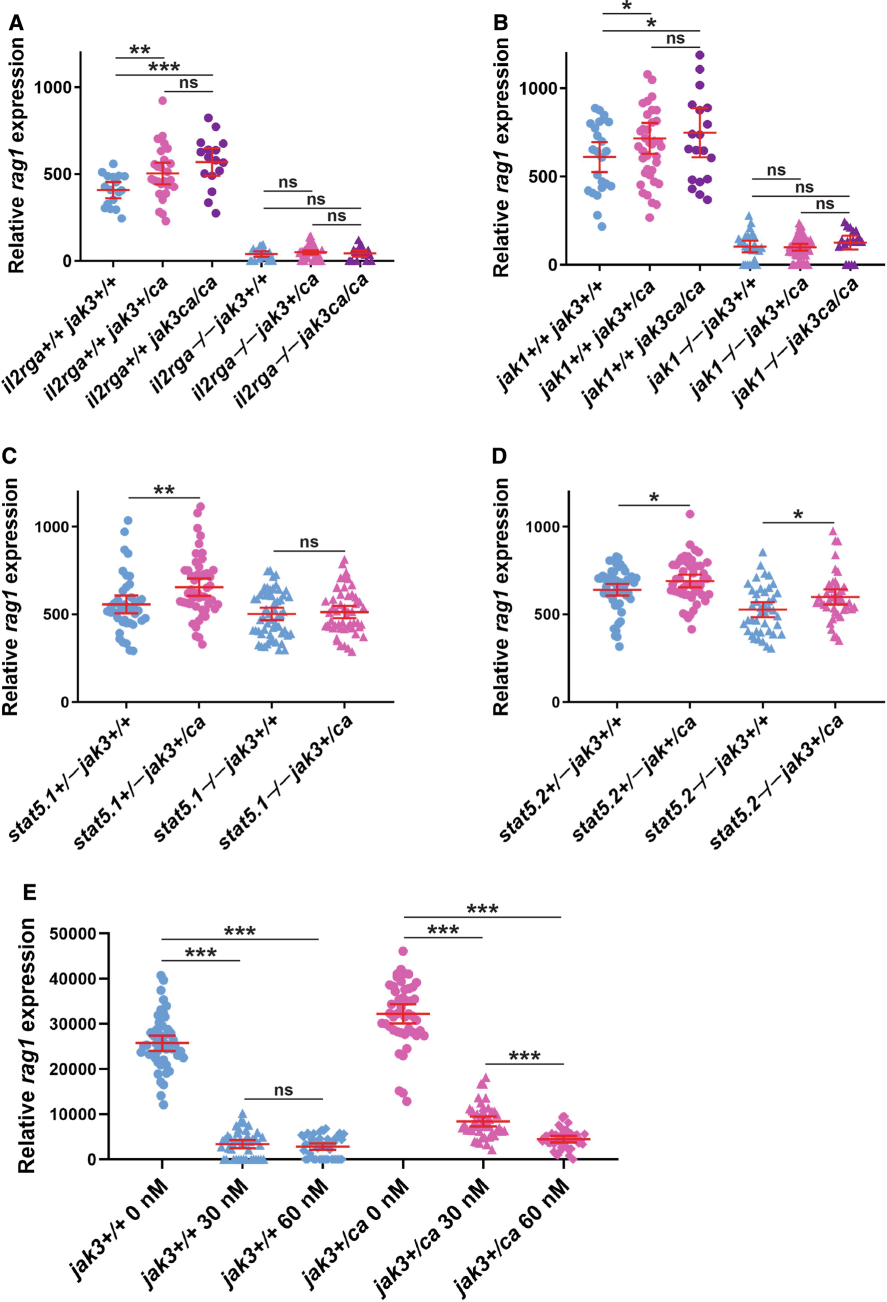
Molecular analysis of Jak3 A573V mutants. A-D. Genetic studies. Relative *rag1* expression of *jak3*^+*/*+^*, jak3*^+*/ca*^ and *jak3*^*ca/ca*^ embryos on the indicated *il2rga* (**A**), *jak1* (**B**), *stat5.1* (**C**) and *stat5.2* (**D**) genetic backgrounds, as detailed in Supp. Figure 1, 2, 3 and 4, respectively. Results for individual embryos of the relevant genotypes are shown along with mean and SEM in red and statistically significant differences indicated (*p < 0.05, **p < 0.01, ***p < 0.001, ns: not significant, *n* = 400). For simplicity, the results for some genotypes in panels **A**, **B** have been omitted. **E** Pharmacological studies. Relative *rag1* expression of *jak3*^+*/*+^*, jak3*^+*/ca*^ and *jak3*^*ca/ca*^ embryos in the presence of the indicated Tofacitib concentrations. Results for individual embryos are shown along with mean and SEM in red and statistically significant differences indicated (***p < 0.001, ns: not significant, *n* = 400)

JAK3 is exclusively associated with the common cytokine receptor chain IL-2Rγc [18]. Zebrafish possesses two paralogues for the human *IL2RG* gene, with *il2rga* shown to have a conserved role in lymphopoiesis acting upstream of *jak3* [59, 60]. Therefore, to elucidate the role of IL-2Rγc in mediating the effects of Jak3 A573V, fish carrying *jak3*^*ca*^ alleles were crossed with those carrying knockout alleles for *il2rga* [59] to produce *il2rga*^+/−^ *jak3*^+*/ca*^ fish that were in-crossed to generate progeny that were assessed for *rag1* expression and genotyped in parallel (Supp. Figure 1). As expected, for those embryos on an *il2rga*^+/+^ background, *rag1* expression was significantly greater in *jak3*^+*/ca*^ and *jak3*^*ca/ca*^ compared to *jak3*^+/+^ embryos (Fig. 4A). For those on an *il2rga*^−/−^ background, relative *rag1* expression was significantly decreased for all *jak3* genotypes, but importantly, there was no longer a significant difference between *jak3*^+/+^ and either *jak3*^+/ca^ or *jak3*^ca/ca^ embryos. This suggests an essential requirement for IL-2Rγc in mediating the effects of JAK3 A573V in vivo.

Amongst IL-2R family members, the ligand-specific receptor chains are associated with JAK1, which has been shown to be essential for signaling [20]. Zebrafish possesses a single *jak1* orthologue [37], which has been demonstrated to play a conserved role in early lymphopoiesis [23]. Therefore, to evaluate the role of JAK1 in JAK3 A573V-mediated lymphoproliferation, *jak3*^*ca*^-carrying fish were crossed with *jak1* mutant fish to produce *jak1*^+/−^ *jak3*^+*/ca*^ fish that were in-crossed to generate progeny for analysis (Supp. Figure 2). For those on a *jak1*^+/+^ background, relative *rag1* expression was greater in *jak3*^+/ca^ and *jak3*^ca/ca^ embryos compared to *jak3*^+/+^ controls to a statistically significant extent (Fig. 4B). However, for those on a *jak1*^−/−^ background, *rag1* expression was significantly decreased for all *jak3* genotypes and notably there was no significant difference observed between *jak3*^+/+^ and either *jak3*^+/ca^ or *jak3*^ca/ca^ embryos, confirming that JAK1 is also required for JAK3 A573V to exert its in vivo impacts.

STAT5 is strongly activated by JAK3 following stimulation of IL-2Rγc-containing receptors [34, 43], and is also activated by JAK3 mutants [17, 41]. Zebrafish possesses two STAT5 proteins encoded by *stat5.1* and *stat5.2* [33], both of which have been implicated downstream of *il2rga/jak3* [60]. Since no differences between *jak3*^+/ca^ and *jak3*^ca/ca^ embryos had thus far been seen, a simpler crossing strategy was used, such that relative *rag1* expression was compared between *jak3*^+/+^ and *jak3*^+/ca^ genotypes on either a *stat5.1* or *stat5.2* homozygote mutant background compared to respective heterozygotes (Supp. Figures 3 and 4). As was the case on a wild-type background, a significant increase in *rag1* was observed in *jak3*^+/ca^ compared to *jak3*^+/+^ embryos on a heterozygote *stat5.1*^+/−^ background embryos (Fig. 4C). However, no statistical difference in *rag1* expression was observed in *jak3*^+/ca^ compared to *jak3*^+/+^ embryos on a *stat5.1*^−/−^ background. In contrast, a significant increase in *rag1* was observed in *jak3*^+/ca^ compared to *jak3*^+/+^ embryos on both *stat5.2*^+/−^ and *stat5.2*^−/−^ backgrounds (Fig. 4D). This suggests a differential requirement for STAT5 paralogues in mediating the effects of JAK3 A573V.

Several JAK inhibitors are under investigation for the treatment of hematopoietic diseases, including myeloproliferative neoplasms and autoimmune diseases, including the JAK3-specific Tofacitinib for treatment of rheumatoid arthritis [8, 27]. The efficacy of Tofacitinib was tested on *jak3*^+*/ca*^ in comparison to *jak3*^+*/*+^ embryos with treatment from 3 to 5 dpf followed by analysis of relative *rag1* staining (Fig. 4E). A significant decrease in *rag1* expression was seen in *jak3*^+*/*+^ embryos treated with 30 nM and 60 nM Tofacitinib compared to the untreated group, with no significant difference between these concentrations, as described previously [60]. The extent of *rag1* staining was also reduced in *jak3*^+*/ca*^ following Tofacitinib treatment, although there was an even greater decrease with 60 nM compared to 30 nM. Collectively, these data show that the Jak3 A573V mutant was susceptible to Tofacitinib, although may require a higher dose for maximal inhibition.

## Discussion

Lymphoid development relies heavily on the IL-2R family of cytokine receptors, which includes IL-2R, IL-4R, IL-7R, IL-9R, IL-15R and IL-21R [31]. Each of these share the common IL-2Rγc that signals via JAK3, as well as a ligand-specific chain that signals through JAK1 and sometimes a third receptor chain, with STAT5 a key downstream effector [30, 36]. Zebrafish has been employed extensively to study blood and immune cell development and its disruption in malignancy [46, 51, 55]. Importantly, it possesses many of the constituent chains of the IL-2R family, including a duplicated IL-2Rγc [38], as well as homologues of JAK1, JAK3 and STAT5 proteins [33, 37], with conserved roles described for IL-2Rγc, JAK1, JAK3 and STAT5 in embryonic lymphopoiesis [23, 60]. Constitutively activating mutations in JAK3 (or JAK1) are associated with a range of hematological malignancies [3, 6, 19, 44, 48], with the PKD mutations A572V and A573V common in several forms of disease [3, 6, 27, 49, 64]. Since the sequence around A572/A573 was highly conserved, these specific mutations could be readily recapitulated in the zebrafish protein. The zebrafish Jak3 A573V mutant elicited greater activation of zebrafish Stat5.1 in cell culture than the A572V mutant and was also able to significantly increase T cells when introduced into zebrafish embryos. Therefore, to study the impact of enhanced Jak3 activation in a physiologically-relevant context across the life course, a zebrafish Jak3 A573V mutant line was generated using CRISPR–Cas9. This mutant was broadly characterized during embryonic, larval and adult hematopoiesis, with its genetic interaction with other IL-2R family signaling components and sensitivity to a JAK inhibitor analyzed.

The impacts of the Jak3 A573V mutation were evident already in early lymphoid precursors in the developing embryo through elevated *ikzf1* staining at 3.5 dpf. This continued at 5 dpf, when the more mature T cell markers *rag1*, *tcra* and *lck* were also significantly elevated, with analysis of blood smears confirming increased numbers of circulating lymphocytes. Elevated T and NK cell markers were evident in juvenile fish at 28 dpf, but no significant effects on B cells were observed. Adult Jak3 A573V mutants also possessed increased lymphocytes in the blood and kidney, with elevated T and NK cells confirmed in adult male kidney. It has been suggested that the presence of wild-type JAK3 suppresses the effects of mutant JAK3s, with patient *JAK3* mutations often being homozygous or compound heterozygous [16, 40]. However, zebrafish carrying two copies of the allele encoding Jak3 A573V mutant did not show more severe impacts on lymphopoiesis than those with one copy, although reduced survival of homozygote mutants compared to wild-type fish was observed that was not seen with heterozygotes. This could reflect a tendency to progress to a more severe disorder, although no evidence of overt leukemia has been identified. Together these data are consistent with constitutively active JAK3 eliciting a mild lymphoproliferative disorder rather than overt leukemia, with mutation of a single allele sufficient to achieve this.

Mouse transplantation models have been widely used to study JAK3 mutants. For example, retroviral transduction of mouse bone marrow cells to overexpress human JAK3 A572V followed by transplantation into lethally irradiated mice resulted in megakaryocytic hyperplasia and lymphoproliferative disorders [11], including a T-ALL like disease, with ligand-independent proliferation of T cells [17]. However, these studies involved overexpression of the mutant JAK3 across multiple cell lineages also expressing wild-type mouse Jak3, in contrast to introducing the mutation into the endogenous protein expressed from its native promoter within its normal cellular context. Notably, a mouse Jak3 A572V knock-in model exhibited a more similar phenotype to the zebrafish model, with progressive expansion of CD8+ T cells and minor skin pathology, with a fully penetrant, lethal disease only manifest in cooperation with partial trisomy 21 [57]. These more physiologically relevant models emphasize the importance of additional co-operating mutations for leukemic transformation, as is observed in humans [3, 6, 17]. Key co-operating mutations include those in genes encoding the epigenetic regulators SUZ12 [9] and PHF6 [69] as well as the transcription factors RUNX1 [2] and HOXA9 [15], with the latter co-occupying similar genomic loci as STAT5. One explanation for the absence of significant B cell involvement in either model is that while JAK3 is a ‘driver’ mutation in T cell malignancy, it is considered more of a ‘co-operating’ gene in B cell malignancy, as described in the context of *PAX5* fusions [24] and *SPI1* deletions [5]. Crossing the zebrafish Jak3 A573V mutant with lines harboring other oncogenic mutations would yield definitive insights.

Previous research has demonstrated that most JAK3 mutants require IL-2Rγc to activate downstream signaling and factor-independent growth [1], and both IL-2Rγc and JAK1 for maximal activation of downstream signaling [17, 41, 54]. The kinase activity of JAK1 has been shown to be essential to facilitate this [20, 41, 54], with JAK1 constitutively activated in the presence of JAK3 mutants [16, 41]. In this study, genetic analysis has revealed that the requirement for IL-2Rγc and JAK1 extends to the in vivo environment, with both found to be essential for mutant JAK3 to mediate the expansion of embryonic T lymphocytes. Such a role for IL-2Rγc has also been observed in the mouse Jak3 A572V knock-in model [57]. These data are consistent with JAK3 mutations occurring in concert with JAK1 mutations [3, 6, 19, 49], with JAK1 and JAK3 mutations able to synergize in vitro [20].

STAT5 proteins are constitutively activated by JAK3 mutants [16, 41]. The data presented here demonstrates for the first time a requirement for downstream STAT5 in mediating the effects of JAK3 mutants—and specifically the zebrafish Stat5.1 paralogue, which has been identified as the functional equivalent of STAT5B rather than STAT5A [68]. These results are compatible with studies showing STAT5B exerting a greater effect on cytokine-driven lymphocyte proliferation than STAT5A [22, 47]. Moreover, activating STAT5B mutations have been identified in a variety of hematological neoplasms, being identified as likely driver mutations in various T cell neoplasms [14, 52, 63], with JAK3 and STAT5B in the same mutation cluster in ALL [40]. Interestingly STAT5B but not STAT5A has also been demonstrated to facilitate BCR/ABL-induced leukemic transformation [26], and we have previously shown that a zebrafish Stat5.1 N649H mutant can reproduce features of STAT5B N649H-mediated disease that impacts both lymphoid and myeloid lineages [32]. Collectively, this highlights a specific, and evolutionarily conserved role for STAT5B/Stat5.1 in hematological malignancy.

The identification of JAK mutations in a broad range of hematological malignancies and proliferative disorders has promoted the development of a myriad of pharmacological inhibitors [42, 53]. In this study, we show that Jak3 A573V-mediated lymphocyte expansion was sensitive to Tofacitib, a pan-JAK inhibitor shown to be efficacious in human clinical trials [12]—confirming similar results in the mouse Jak3 A572V model [57] and a variety of in vitro studies [8, 16, 27]. In fact, one of these studies identified STAT5 activation as showing the highest sensitivity to the effects of this inhibitor [16], further emphasizing its key role. Together this not only serves to confirm the applicability of this model to pharmacological testing, but also indicates that approaches targeting STAT5B would also be appropriate to consider.

## Conclusion

This study demonstrated that a single JAK3 A573V allele expressed from its endogenous locus was able to enhance zebrafish lymphopoiesis throughout the life-course, producing a sustained increase in T cells, as well as affecting NK cells, but not B cells. This was mediated via an IL-2Rγc/JAK1/JAK3/STAT5B signaling pathway and was sensitive to Tofacitinib. This enhances our understanding of oncogenic JAK3 mutations in vivo and creates a novel model to underpin further translational investigations.

## Supplementary Information

Below is the link to the electronic supplementary material.

Supplementary Figure 1: Investigating the role of IL-2Rγc in mediating the effects of Jak3 A573V. A. Embryos were raised from *il2rga*^+/−^* jak3*^+/ca^ × *il2rga*^+/−^* jak3*^+/ca^ crosses until 5 dpf then subjected to WISH with *rag1*. Individual embryos were imaged to determine the area of *rag1* expression and then genomic DNA was extracted for PCR-based genotyping, using HRM analysis for *jak3* (lower left) and RFLP analysis for *il2rga* (lower right) using a PCR with *il2rga*-specific primers (5’- CGAAGACTGTCCTGAATATGAGAC, 5’- TCTGGTCAGTCCTGTAACGAAC) followed by *Nde*I digestion. B-G. Images of *rag1* staining for representative *il2rga*^+/+^* jak3*^+/+^ (B), *il2rga*^−/−^* jak3*^+/+^ (C), *il2rga*^+/+^* jak3*^+/ca^ (D), *il2rga*^−/−^* jak3*^+/ca^ (E), *il2rga*^+/+^* jak3*^ca/ca^ (F) and *il2rga*^−/−^* jak3*^ca/ca^ (G) embryos with *rag1* expression indicated by arrows (JPG 7059 kb)

Supplementary Figure 2: Investigating the role of Jak1 in mediating the effects of Jak3 A573V. A. Embryos were raised from *jak1*^+/−^* jak3*^+/ca^ × *jak1*^+/−^* jak3*^+/ca^ crosses until 5 dpf then subjected to WISH with *rag1*. Individual embryos were imaged to determine the area of *rag1* expression and then genomic DNA was extracted for PCR-based genotyping, using HRM analysis for *jak3* (lower left) and *jak1* (lower right) with *jak1*-specific primers (5’-CAGGCATTCTTTGAGACCGC, 5’-GGGTACTTACTCTCCTGGTGACG). B-G. Images of *rag1* staining for representative *jak1*^+/+^* jak3*^+/+^ (B), *jak1*^−/−^* jak3*^+/+^ (C), *jak1*^+/+^* jak3*^+/ca^ (D), *jak1*^−/−^* jak3*^+/ca^ (E), *jak1*^+/+^* jak3*^ca/ca^ (F) and *jak1*^−/−^* jak3*^ca/ca^ (G) embryos with *rag1* expression indicated by arrows (JPG 7856 kb)

Supplementary Figure 3: Investigating the role of Stat5.1 in mediating the effects of Jak3 A573V. A. Embryos were raised from a *stat5.1*^+/−^* jak3*^+/ca^ × *stat5.1*^−/−^* jak3*^+/+^ cross until 5 dpf then subjected to WISH with *rag1*.Embryos were imaged to determine the area of *rag1* expression and then genomic DNA was extracted for PCR-based genotyping, using HRM analysis for *jak3* (lower left) and PCR for *stat5.1* (lower right) with *stat5.1*-specific primers (5’-GTGGGCGGGTTAATGGACAG, 5’-TACACGCATACCCTGTATTCTGAG). B-E: Images of *rag1* expression for representative *stat5.1*^+/−^ *jak3*^+/+^ (B), *stat5.1*^+/−^ *jak3*^+/ca^ (C), *stat5.1*^−/−^ *jak3*^+/+^ (D), and *stat5.1*^−/−^ *jak3*^+/ca^ (E) embryos with *rag1* expression indicated by arrows (JPG 4091 kb)

Supplementary Figure 4: Investigating the role of Stat5.2 in mediating the effects of Jak3 A573V. A. Embryos were raised from a *stat5.2*^+/−^* jak3*^+/ca^ × *stat5.2*^−/−^* jak3*^+/+^ cross until 5 dpf then subjected to WISH with *rag1*.Embryos were imaged to determine the area of *rag1* expression and then genomic DNA was extracted for PCR-based genotyping, using HRM analysis for *jak3* (lower left) and PCR for *stat5.2* (lower right) with *stat5.2*-specific primers (5’- CAGCAGTCCAGGTTCAGGTC, 5’-GATCATACCCTGTATCCTCAAACTC). B-E: Images of *rag1* expression for representative *stat5.2*^+/−^ *jak3*^+/+^ (B), *stat5.2*^+/−^ *jak3*^+/ca^ (C), *stat5.2*^−/−^ *jak3*^+/+^ (D), and *stat5.2*^−/−^ *jak3*^+/ca^ (E) embryos with *rag1* expression indicated by arrows (JPG 5797 kb)

## Data Availability

All data generated or analyzed during this study are included in this published article (and its supplementary information files).

## Abbreviations

AMKL: Acute megakaryoblastic leukemia
ca: Constitutively activating
Cas9: CRISPR-associated 9
CRISPR: Clustered regularly interspaced short palindromic repeats
CTCL: Cutaneous T cell leukemia
dpf: Days post-fertilization
FSC: Forward scatter
HDR: Homology-directed repair
HRM: High-resolution melt
IL: Interleukin
IL-2Rγc: IL-2 receptor gamma common
JAK: Janus kinase
NKTCL: Natural killer T cell lymphoma
PKD: Pseudokinase domain
qRT^2^-PCR: Quantitative real-time RT-PCR
RFLP: Restriction fragment length polymorphism
RT-PCR: Reverse transcription-polymerase chain reaction
SEM: Standard error of the mean
SSC: Side scatter
STAT: Signal transducer and activator of transcription
T-ALL: T cell acute lymphoblastic leukemia
T-PLL: T cell prolymphocytic leukemia
WISH: Whole-mount in situ hybridization
