# Supplementary figures and images for: In vivo impact of JAK3 A573V mutation revealed using zebrafish
Source: Cell Mol Life Sci. 2022 May 27;79(6):322. doi: 10.1007/s00018-022-04361-8 (PMC9142468; doi:10.1007/s00018-022-04361-8)

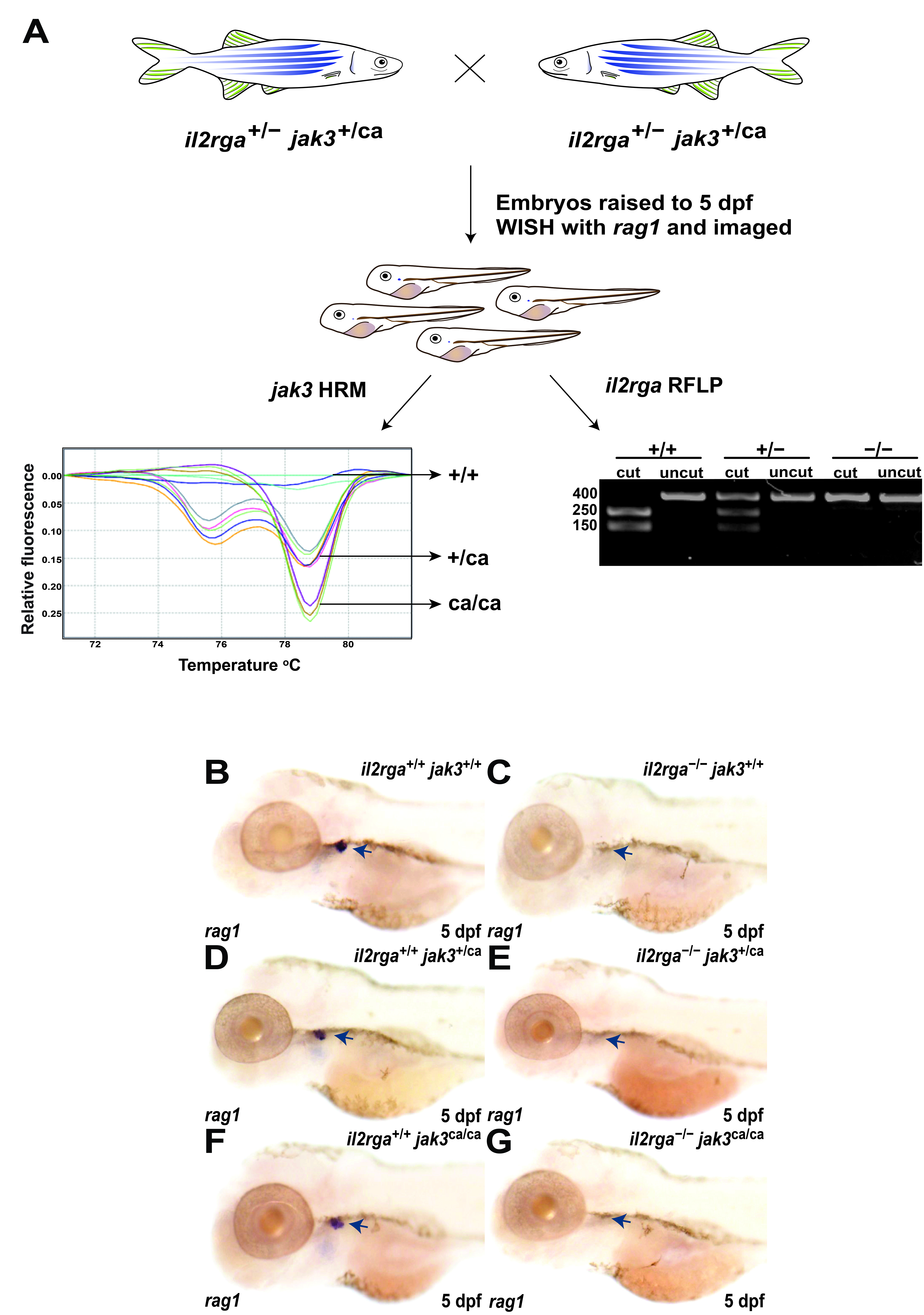

Supplement: Supplementary file 1 — Supplementary Figure 1: Investigating the role of IL-2Rγc in mediating the effects of Jak3 A573V. A. Embryos were raised from il2rga+/− jak3+/ca × il2rga+/− jak3+/ca crosses until 5 dpf then subjected to WISH with rag1. Individual embryos were imaged to determine the area of rag1 expression and then genomic DNA was extracted for PCR-based genotyping, using HRM analysis for jak3 (lower left) and RFLP analysis for il2rga (lower right) using a PCR with il2rga-specific primers (5’- CGAAGACTGTCCTGAATATGAGAC, 5’- TCTGGTCAGTCCTGTAACGAAC) followed by NdeI digestion. B-G. Images of rag1 staining for representative il2rga+/+ jak3+/+ (B), il2rga−/− jak3+/+ (C), il2rga+/+ jak3+/ca (D), il2rga−/− jak3+/ca (E), il2rga+/+ jak3ca/ca (F) and il2rga−/− jak3ca/ca (G) embryos with rag1 expression indicated by arrows (JPG 7059 kb) [file 18_2022_4361_MOESM1_ESM.jpg]

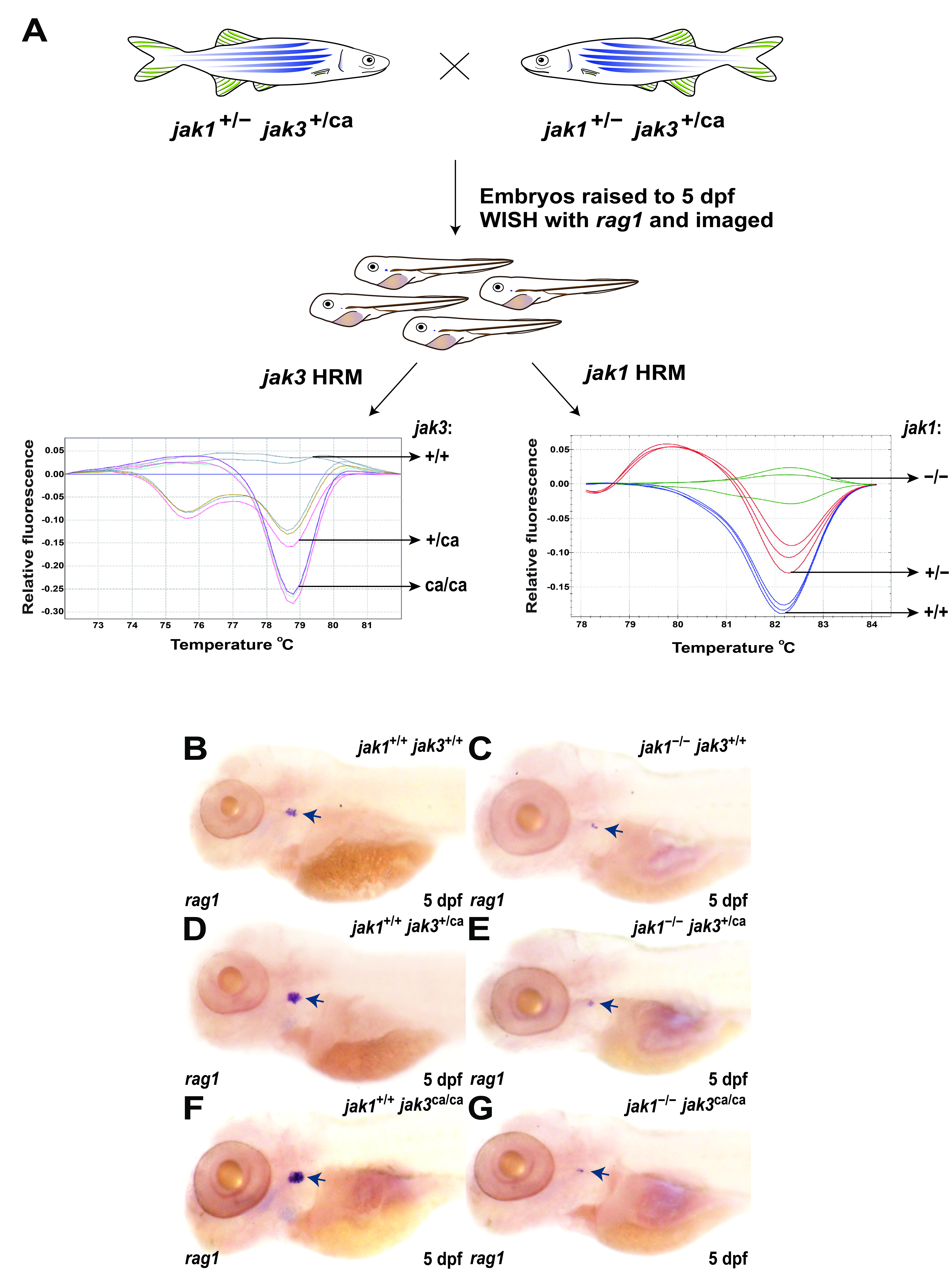

Supplement: Supplementary file 2 — Supplementary Figure 2: Investigating the role of Jak1 in mediating the effects of Jak3 A573V. A. Embryos were raised from jak1+/− jak3+/ca × jak1+/− jak3+/ca crosses until 5 dpf then subjected to WISH with rag1. Individual embryos were imaged to determine the area of rag1 expression and then genomic DNA was extracted for PCR-based genotyping, using HRM analysis for jak3 (lower left) and jak1 (lower right) with jak1-specific primers (5’-CAGGCATTCTTTGAGACCGC, 5’-GGGTACTTACTCTCCTGGTGACG). B-G. Images of rag1 staining for representative jak1+/+ jak3+/+ (B), jak1−/− jak3+/+ (C), jak1+/+ jak3+/ca (D), jak1−/− jak3+/ca (E), jak1+/+ jak3ca/ca (F) and jak1−/− jak3ca/ca (G) embryos with rag1 expression indicated by arrows (JPG 7856 kb) [file 18_2022_4361_MOESM2_ESM.jpg]

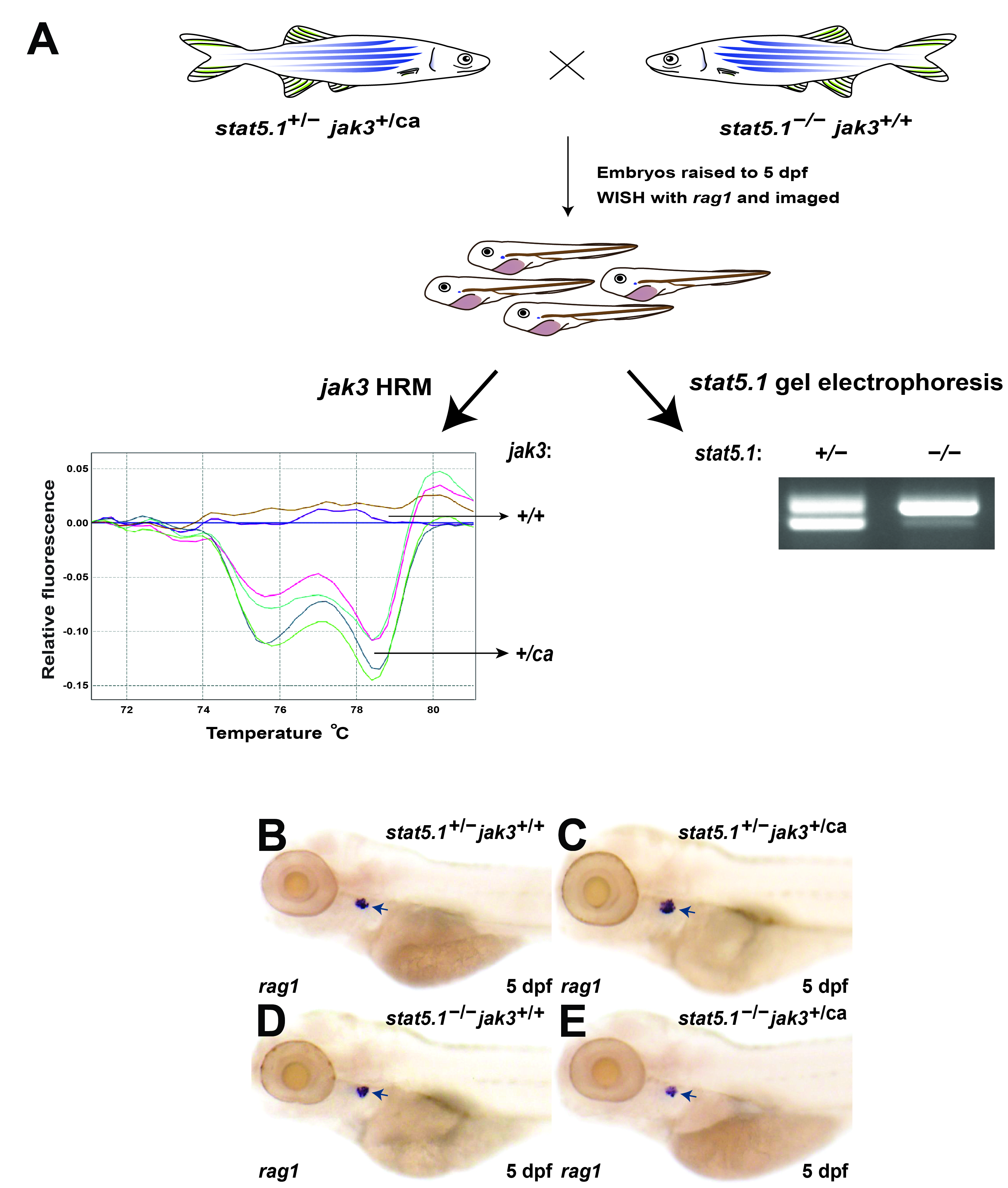

Supplement: Supplementary file 3 — Supplementary Figure 3: Investigating the role of Stat5.1 in mediating the effects of Jak3 A573V. A. Embryos were raised from a stat5.1+/− jak3+/ca × stat5.1−/− jak3+/+ cross until 5 dpf then subjected to WISH with rag1.Embryos were imaged to determine the area of rag1 expression and then genomic DNA was extracted for PCR-based genotyping, using HRM analysis for jak3 (lower left) and PCR for stat5.1 (lower right) with stat5.1-specific primers (5’-GTGGGCGGGTTAATGGACAG, 5’-TACACGCATACCCTGTATTCTGAG). B-E: Images of rag1 expression for representative stat5.1+/− jak3+/+ (B), stat5.1+/− jak3+/ca (C), stat5.1−/− jak3+/+ (D), and stat5.1−/− jak3+/ca (E) embryos with rag1 expression indicated by arrows (JPG 4091 kb) [file 18_2022_4361_MOESM3_ESM.jpg]

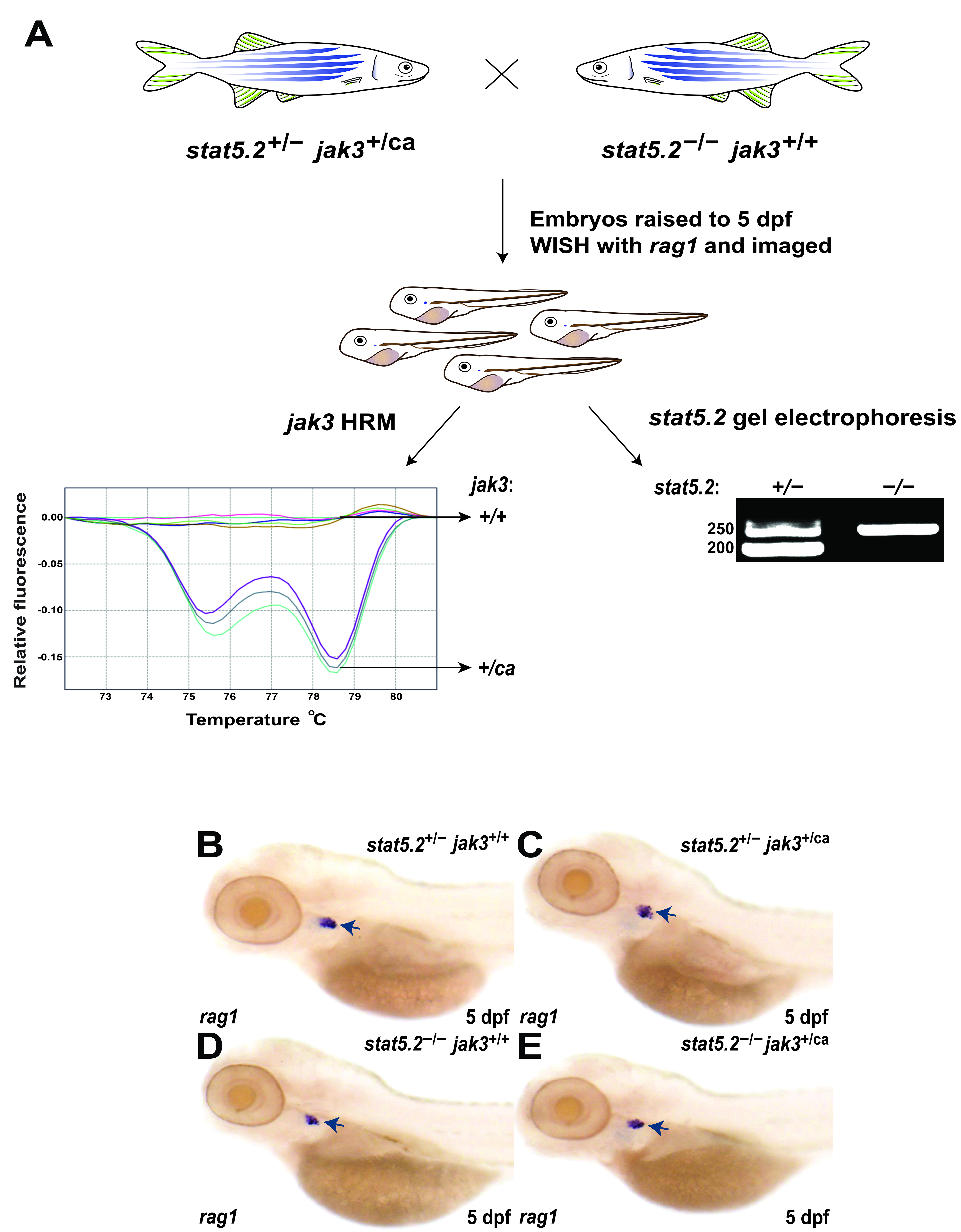

Supplement: Supplementary file 4 — Supplementary Figure 4: Investigating the role of Stat5.2 in mediating the effects of Jak3 A573V. A. Embryos were raised from a stat5.2+/− jak3+/ca × stat5.2−/− jak3+/+ cross until 5 dpf then subjected to WISH with rag1.Embryos were imaged to determine the area of rag1 expression and then genomic DNA was extracted for PCR-based genotyping, using HRM analysis for jak3 (lower left) and PCR for stat5.2 (lower right) with stat5.2-specific primers (5’- CAGCAGTCCAGGTTCAGGTC, 5’-GATCATACCCTGTATCCTCAAACTC). B-E: Images of rag1 expression for representative stat5.2+/− jak3+/+ (B), stat5.2+/− jak3+/ca (C), stat5.2−/− jak3+/+ (D), and stat5.2−/− jak3+/ca (E) embryos with rag1 expression indicated by arrows (JPG 5797 kb) [file 18_2022_4361_MOESM4_ESM.jpg]
